# Development of Human Mast Cells from Hematopoietic Stem Cells within a 3D Collagen Matrix: Effect of Stem Cell Media on Mast Cell Generation

**DOI:** 10.1155/2018/2136193

**Published:** 2018-07-11

**Authors:** Tahereh Derakhshan, Rudra Bhowmick, Jerry W. Ritchey, Heather Gappa-Fahlenkamp

**Affiliations:** ^1^School of Chemical Engineering, Oklahoma State University, 420 Engineering North, Stillwater, OK 74078, USA; ^2^Department of Veterinary Pathobiology, Center for Veterinary Health Sciences, Oklahoma State University, 250 McElroy Hall, Stillwater, OK 74078, USA

## Abstract

Mast cells (MCs) arise from hematopoietic stem cells (HSCs) that mature within vascularized tissues. Fibroblasts and endothelial cells (ECs) play a role in the maturation of HSCs in the tissues. Due to difficulties in isolating MCs from tissues, large numbers of committed MC precursors can be generated in 2D culture systems with the use of differentiation factors. Since MCs are tissue-resident cells, the development of a 3D tissue-engineered model with ancillary cells that more closely mimics the 3D *in vivo* microenvironment has greater relevance for MC studies. The goals of this study were to show that MCs can be derived from HSCs within a 3D matrix and to determine a media to support MCs, fibroblasts, and ECs. The results show that HSCs within a collagen matrix cultured in StemSpan media with serum added at the last week yielded a greater number of c-kit^+^ cells and a greater amount of histamine granules compared to other media tested. Media supplemented with serum were necessary for EC survival, while fibroblasts survived irrespective of serum with higher cell yields in StemSpan. This work demonstrates the development of functional MCs within a 3D collagen matrix using a stem cell media that supports fibroblast and ECs.

## 1. Introduction

Release of preformed mediators and expression of diverse molecules have placed mast cells (MCs) among the foremost inducers of allergic responses and regulators of innate and adaptive immunity [1, 2]. MCs are abundant in tissue near surfaces exposed to the external environment, and their number and distribution change markedly during immune responses [3–5]. During immunoglobulin E- (IgE-) dependent responses, cross-linking of the Fc*ε*RI/IgE complexes leads to MC activation and degranulation of a wide range of bioactive products, including histamine [2]. The mediators increase vascular permeability and recruit leukocytes to the site of insult, resulting in hyperemia and edema, the cardinal symptoms of acute inflammation [6]. MC mediators can exert multiple effects, such as extracellular matrix remodeling in fibrosis or degradation during early events of angiogenesis, giving them the potential to be involved in the pathogenesis of a variety of disorders [7–9].

Although MCs are not normally present in circulation, they can be obtained from progenitor cells in the presence of T cell-derived cytokines and fibroblast-derived stem cell factor (SCF) [10–12]. CD133 and CD34 antigens are markers for primitive progenitor and hematopoietic stem cell (HSC) populations [13, 14]. Previous studies have shown that upon treatment with SCF, interleukin- (IL-) 3, and IL-6, CD133^+^ HSCs isolated from various sources, including cord blood and peripheral blood, can differentiate into MCs under a two-dimensional (2D) culture condition [15, 16]. The growth, phenotype, and function of the generated MCs can be altered by the culture media supplements [17–19]. As an instance, the addition of serum to the media from the beginning of culture can result in a low number of mature MCs with reduced Fc*ε*RI expression, while its addition at later weeks of the culture period promotes the expression of Fc*ε*RI and histamine release upon activation [20, 21]. Although the generation of MCs under 2D culture conditions provided a source for human MC studies, they have been considered as immature MCs or “incomplete representatives of mature MCs” due to the lack of *in vivo* microenvironmental conditions that may affect MC phenotypic and functional characteristics [1, 22]. Since MCs mature and interact with other cells within tissue, providing a condition that better mimics the *in vivo* three-dimensional (3D) milieu would be of greater relevance for studying MC responses and immunoregulatory roles. In fact, interaction between MCs and extracellular matrix components can affect MC behavior and influence their biological functions [23]. Therefore, the first objective of this study was to demonstrate the generation of MCs within a 3D collagen matrix, which provides the conditions for investigating the cellular interactions that are not possible to examine within a conventional 2D culture system.

MCs are located near blood or lymphatic vessels in proximity to fibroblasts that are a principal cellular component of tissue [22]. Previous studies have shown that the cross talk between MCs, fibroblasts, and endothelial cells (ECs) mediates various physiological and pathological processes [24, 25]. Besides the release of growth factors that are essential for MC survival and maturity, direct interaction between fibroblasts and ECs can regulate MC development [26–28]. Therefore, incorporation of fibroblasts and ECs into the 3D tissue model allows the transmission of similar signaling molecules that HSCs may receive during differentiation into MCs from neighboring cells *in vivo*. However, for the creation of such a tissue-engineered model, there are no universal media that support the overall growth of MC precursors and the abovementioned ancillary cells. In fact, selecting the appropriate culture media is determinative to the success of a coculture system. Since each cell type has specific growth requirements, a suitable media that regulates their survival needs to be determined. Previous studies have shown the expansion of CD34^+^ HSCs or their differentiation into MCs by using cytokine-supplemented and serum-free media [29–32]. However, most studies with fibroblasts and ECs use media with serum not specific for HSC growth and differentiation [33–35]. Therefore, the second objective of the current study was to determine a medium that would support the generation of functional MCs from HSCs, as well as one that would support the normal characteristics of fibroblasts and ECs. Having an understanding of the effect of culture media on each individual cell type is an important first step needed before the development of a coculture model with multiple cell interactions.

## 2. Material and Methods

### 2.1. Antibodies and Reagents

M199 and StemPro®-34 SFM culture media were purchased from Life Technologies (Carlsbad, CA). HPGM™ and StemSpan™ SFEM cell culture media were purchased from Lonza (Walkersville, MD) and STEMCELL Technologies (Vancouver, Canada), respectively. Human SCF, IL-6, and IL-3 were purchased from ProSpec (Rehovot, Israel) or PeproTech (Rocky Hill, NJ). Defined HyClone fetal bovine serum (FBS) was purchased from GE Healthcare Life Sciences (Logan, UT). Human fibroblasts treated with mitomycin C were purchased from Merck Millipore (Billerica, MA), while human umbilical vein ECs were purchased from PromoCell (Heidelberg, Germany). Anti-human fluorochrome-conjugated CD117/c-kit (clone 104D2), Fc*ε*RI (clone CRA-1), and CD31 (clone WM59), and their isotype controls, Ms IgG1 (clone MOPC-21) and Ms IgG2b (clone MPC-11), were purchased from BioLegend (San Diego, CA). Anti-human fluorochrome-conjugated CD90 (clone 5E10), its isotype, Ms IgG1 (clone MOPC-21), and mouse anti-human tryptase (clone AA1) were purchased from Abcam (Cambridge, MA). Mouse anti-human chymase (clone B7) and the secondary antibody goat anti-mouse IgG1 were from Chemicon International Inc. (Temecula, CA) and Santa Cruz Biotechnology (Dallas, TX), respectively.

### 2.2. Cell Culture

#### 2.2.1. Hematopoietic Stem Cell (HSC) Culture

CD133^+^ cells were obtained from human peripheral blood mononuclear cells (PBMCs). PBMCs were isolated from fresh leukocyte preparations (obtained from the Oklahoma Blood Institute; Oklahoma City, OK) by the Ficoll-Paque density separation method (GE Healthcare; Pittsburgh, PA). CD133^+^ cells were isolated from PBMCs using a magnetic separation kit (MACS Miltenyi Biotec; Bergisch Gladbach, Germany).

CD133^+^ cells were cultured and differentiated within a 3D collagen gel. For this, a 2 mg/ml collagen solution was prepared by modifying a previous protocol [36] using 64.5 vol% of 3.1 mg/ml type 1 bovine collagen (Advanced BioMatrix, Carlsbad, CA), 8.1 vol% 10x M199, 13.3 vol% 0.1 N NaOH, and 14.1 vol% PBS. CD133^+^ cells were mixed with the collagen solution (5.1 × 10^5^ cells/ml) and added to the cell culture plates. Following gel formation (45 min), media were added to the samples and the samples were incubated for seven weeks with media changes once a week. Media were supplemented with human SCF (100 ng/ml), IL-6 (50 ng/ml), and IL-3 (1 ng/ml) for the first three weeks of culture. The CD133^+^ cells were monitored weekly and characterized on the seventh week of culture. In order to study the effect of serum on the growth and differentiation of CD133^+^ cells, FBS (10%, v/v) was added at three different time points: (i) from the day of seeding until the end of the seventh week (Ser1–7), (ii) on the seventh week only (Ser7), and (iii) on the first, second, and seventh week (Ser1, 2, and 7). In all cases, media was changed once a week.

#### 2.2.2. Fibroblast and Endothelial Cell (EC) Culture

Cell culture plates (24 wells, area 1.9 cm^2^, Greiner Bio-One, Monroe, NC) were coated with fibronectin (25 *μ*g/ml in phosphate-buffered saline, PBS) for 2 h before cell seeding. Fibroblasts and ECs were cultured separately in serum-free StemSpan, StemPro, and HPGM. M199 containing 1 vol% PSG (penicillin, streptomycin, and L-glutamine) was used as the “standard media” [33, 34, 37]. ECs were cultured in media with and without the addition of FBS. Fibroblast and ECs were cultured at 35,000 cell/cm^2^ and 12,000 cell/cm^2^ density, respectively, at 37°C, 5% CO_2_ (defined here as “standard conditions”) with media changes on every other day until confluent and ready for testing.

### 2.3. Characterization of Mast Cells (MCs)

#### 2.3.1. Yield and Granule Formation

In order to determine the number of generated viable cells, the collagen matrix was digested after incubation with 2 mg/ml of collagenase D (Roche Applied Science; Indianapolis, IN). The harvested cells in the digested solution were counted by a hemocytometer and the number of viable cells was determined by trypan blue exclusion. The cell yield was calculated as the ratio of the number of viable cells harvested to the number of cells seeded. Cytosolic granule formation was determined by Wright-Giemsa staining using an automated stainer (Ames Hema-Tek Stainer).

#### 2.3.2. Expression of Phenotypic Markers

Expression of c-kit and Fc*ε*RI was assessed by flow cytometry. After seven weeks of culture, the expression of Fc*ε*RI was stabilized by incubating the cells for 24 h with myeloma IgE (2 *μ*g/ml, Merck Millipore). Following collagenase D digestion of the matrix, the cells were collected, stained using anti-c-kit and anti-Fc*ε*RI antibodies or relevant isotype controls, and analyzed by flow cytometry. Dead cells were excluded by PI staining.

For immunocytochemical staining of tryptase and chymase granules, the cells were collected from the matrix and fixed by using a fixation/permeabilization solution kit (BD Biosciences; CA). After incubation with a blocking solution containing 10% goat serum (v/v%, Gibco; CA) for 1 h, the cells were incubated with primary antibodies against tryptase or chymase or isotype control. Following this, a secondary antibody was added, and incubated for 30 min at room temperature. The cells were incubated for at least 1 h in the staining buffer containing 0.2% bovine serum albumin (BSA), prior to staining with anti-c-kit antibody and analysis by flow cytometry.

#### 2.3.3. Activation and Histamine Release

At seven weeks postseeding, the function of the generated cells was examined by cross-linking the Fc*ε*RI receptors by IgE and anti-IgE. Activation was performed for cells within the matrix and for cells removed from the matrix. Cells were sensitized with 15 *μ*g/ml myeloma IgE (Athens Research & Technology; Athens, GA) in complete media for 24 h and rinsed three times prior to activation with various concentrations of anti-IgE (Chemicon International Inc.; Temecula, CA) in Tyrode's solution (Boston BioProducts; Ashland, MA) supplemented with SCF and IL-6 for 1 h. For measuring the cellular histamine, cells were lysed by freeze-thaw cycles in water and sonicated for 5 min. Histamine was quantified using a commercial enzyme-linked immunosorbent assay (ELISA) kit (Labor Diagnostika Nord; Nordhorn, Germany). The percentage of histamine release is determined by taking the ratio of the total amount of histamine released by the cells to the total histamine content initially in the cells. In addition, the spontaneous amount of histamine released by the cells under normal conditions was subtracted from the total amount released. For the samples activated within the matrix, the total amount of histamine released by the cells is determined by measuring the amount of histamine in the media and the gel solution.

### 2.4. Characterization of Fibroblast and Endothelial Cells (ECs)

#### 2.4.1. Proliferation

To measure cell proliferation, fibroblasts and ECs were fluorescently labeled with CellTrace or CellTracker (Life Technologies) prior to culture. The stained cells were harvested by trypsinization, and the fluorescent intensities were measured by flow cytometry. As cells divide, the fluorescent probe is split evenly between the daughter cells and the mean fluorescent intensity (MFI) per cell decreases. As a control for nondividing cells, fibroblasts were stained with CellTrace and analyzed before seeding. Dead cells were stained with propidium iodide (PI, Life Technologies) [38]. All the cell dyes were used following the manufacturer's protocols.

#### 2.4.2. Expression of Surface Receptors and Secretion of Mediators

Expression of CD90 by fibroblasts and CD31 by ECs was determined by flow cytometry. Trypsinized cells were collected and stained with anti-CD90 or anti-CD31 antibodies or their isotype controls (45 min, 4°C). Dead cells were excluded by PI staining. To determine the secretion of SCF and IL-6 by fibroblasts and ECs, culture supernatants were collected and analyzed by commercial ELISA kits (PeproTech).

### 2.5. Statistical Analysis

Experimental results are expressed as mean ± SD of three samples. One-way analysis of variance (ANOVA) was selected to determine significant differences between groups. Tukey's or Student's *t*-test was used for pairwise comparison of groups or between two groups, respectively. A value of *p* < 0.05 was considered significant.

## 3. Results and Discussion

### 3.1. Effect of Culture Media on the Generation of Mast Cells (MCs) from CD133^+^ Hematopoietic Stem Cells (HSCs)

M199, our standard media for EC culture that was also used for fibroblasts, either with serum added from the beginning or in the last week of culture, did not support MC generation and survival, as verified by microscopy, viability, and flow cytometry analyses. From the first week, most cells in all the media, except for HPGM (Ser7), formed colonies as a sign of cell generation.

**Figure 1:**
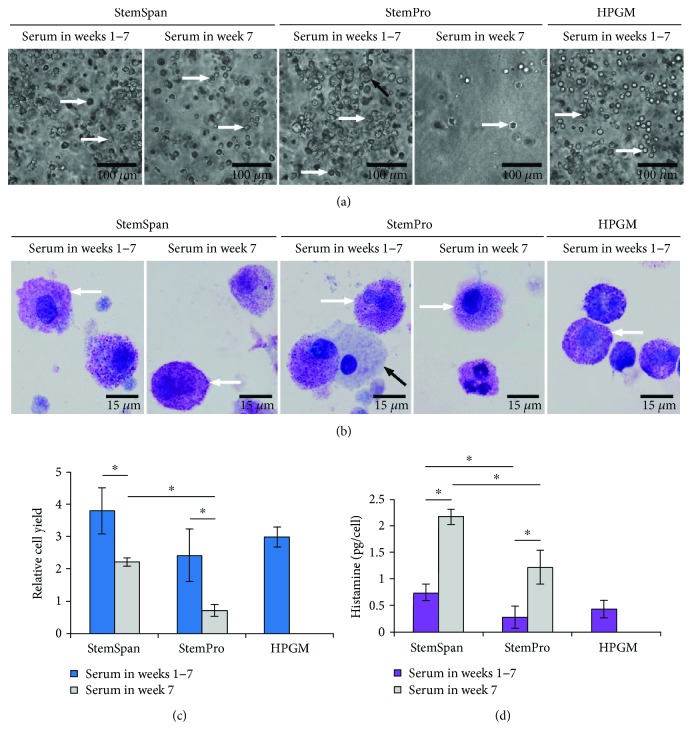
Effect of culture media on the morphology, cell yield, and granule formation of the cells generated from CD133^+^ hematopoietic stem cells (HSCs) in a matrix after seven weeks in culture. (a) Micrographs showing the morphology of generated cells in the test media. White arrows highlight typical MCs. Black arrow highlights the larger-sized MCs found in StemPro (Ser1–7). (b) Metachromatic staining of cytoplasmic granules in the generated cells. A few typical MCs are highlighted by white arrows. In StemPro, some cells were hypogranulated, as shown by the black arrow. (c) Cell yields in the culture media. Cell yield is defined as the ratio of the cells collected at the end of the culture period to the seeded cells. (d) Histamine granule formation in the generated cells. Data are represented as mean ± SD; *n* = 3. ^∗^ indicates *p* < 0.05.

During differentiation, the morphology of MC progenitors sequentially change, until they mature into MCs. Initially, progenitor cells (blasts) have a high nuclear to cytoplasm ratio, and then gradually acquire granules that can be stained to form metachromatic blasts. The atypical type II MCs (called the promastocytes) have bi- or polylobed nuclei, which are oval or eccentrically located, and often possess hypogranulated cytoplasm. At the end of the developmental stage, the mature, typical MCs are formed, which are round or oval with granulated cytoplasm, low nuclear to cytoplasm ratio, and a centrally positioned, round nucleus [39–41]. As shown in Figure 1(a), in the seventh week of culture for all the test media, the cells were mostly round or oval. Except for a few larger cells in the StemPro (Ser1–7) medium, the size of the generated cells in all the test media were in the range of *in vivo* MCs (8–20 *μ*m) [23, 42]. As shown in Figure 1(b), the generated cells exhibited metachromatic cytoplasmic granules following Wright-Giemsa staining, which is a morphological characteristic of MCs [16]. In StemPro (Ser1–7), 30% of the cells were hypogranulated, as shown by the black arrow in Figure 1(b). The results indicate that the cells generated from the MC precursors in the collagen matrix had the morphology of typical, mature and immature MCs, with distinct promastocytic characteristics.

As shown in Figure 1(c), there was no significant difference in cell yields for the media tested with serum from the first week of culture. For the media with serum added in the last week, there was a significantly greater cell yield in StemSpan compared to StemPro (3.1 ± 0.8-fold, *p* < 0.05). For StemSpan (Ser7), the number of cells at termination of culture was 2.2 ± 0.1-fold higher than that of CD133^+^ cells initially seeded in the collagen matrix, which is similar to a 2D culture system that used the same culture medium and generated 3.2 ± 1-fold that of the seeded cells [16].

The histamine content of MCs *in vivo* depends on their anatomic location and subtype. The histamine level in MCs varies from 0.8–12.5 pg/cell to 0.8–5 pg/cell in lung and skin, respectively [42–44]. In this work, the differentiated cells in all but StemPro (Ser1–7) and HPGM media had similar histamine content to that of *in vivo* MCs, and varied between 0.6 and 2.3 pg/cell. Cells cultured in StemPro (Ser1–7) had lower histamine content (0.28 ± 0.21 pg/cell), as verified by the presence of some hypogranulated cells in the Wright-Giemsa-stained sample. MCs generated in StemSpan (Ser7) had significantly greater histamine content compared to all other test media (*p* < 0.05, Figure 1(d)). The histamine content of the MCs generated in a 2D culture system using the StemSpan medium was 15.5 ± 5.3 pg/cell [16], which is higher than the histamine content observed in the *in vivo* MCs. However, this was not the case for the MCs generated within the collagen matrix using the StemSpan medium in this study, with histamine content within the range of *in vivo* observations.

**Figure 2:**
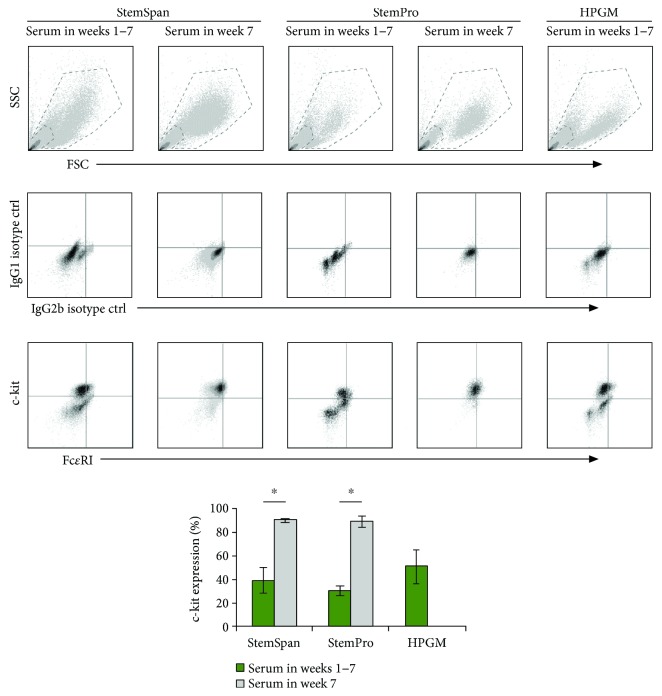
Expression of c-kit and Fc*ε*RI by generated cells from CD133^+^ hematopoietic stem cells (HSCs) in a collagen matrix after seven weeks in culture. The figure shows the gating scheme (top panel) and representative density plots of the isotype control and expression of phenotypic markers (middle and bottom panel, resp.). More than 90% of the cells are gated in the bottom left corner of the isotype density plots. The bar graphs show the percentage of c-kit expression. Data are represented as mean ± SD; *n* = 3. ^∗^ indicates *p* < 0.05.

In addition to morphology and granule formation, immunophenotypical markers are used to distinguish MCs from other cell types. MCs, basophils, eosinophils, dendritic cells, macrophages, and ECs are known to have common precursor cells [45–48], but these cell types vary in their immunophenotype and expression of other markers. MCs, progenitor and HSCs, and ECs all express c-kit [49]. HSCs, ECs, eosinophils, and cells of monocytic lineages lack histamine granules and possess significantly different morphology [50]. Basophils express histamine and Fc*ε*RI, but previous studies have shown that they do not express c-kit [51, 52]. Out of these cell populations, only mature MCs possess histamine granules and express both Fc*ε*RI and c-kit receptors, making these markers useful to define the MC phenotype. As shown in Figure 2, in the media where serum was added at the first week of culture, there was no significant difference in c-kit expression; however, there was a 1.6 ± 0.1- and 1.8 ± 0.1-fold increase in c-kit density (MFI, *p* < 0.05) for StemSpan and HPGM compared to StemPro, respectively. Similarly, for media with serum added in the last week of culture, there was no significant difference in c-kit expression, with 1.3 ± 0.2 times more c-kit density (MFI, *p* < 0.05) in StemSpan compared to StemPro. A 2D culture method that used StemSpan media also showed a high percentage of c-kit-positive cells (88.3 ± 2.2%) [16]. The expression of Fc*ε*RI was similar for all the test media (10–23% on average); however, the Fc*ε*RI density (MFI) was 1.4–2.3 times higher (*p* < 0.01) in StemSpan (Ser1–7) than for the other media. The expression of Fc*ε*RI was lower in comparison with 2D culture systems that used StemSpan or StemPro media [16, 53]. Other reports have shown that incubation with IgE antibody modulates the expression of Fc*ε*RI and can contribute to its detection [54, 55]. For all the test media, IgE was added to the cells in the collagen matrix, prior to collecting the cells and measuring Fc*ε*RI expression. The lower expression could be due to the binding of IgE with the matrix, resulting in less IgE available to interact with the matrix-embedded cells, compared to the other system with cells in suspension.

### 3.2. Effect of Serum on Mast Cell (MC) Development

**Figure 3:**
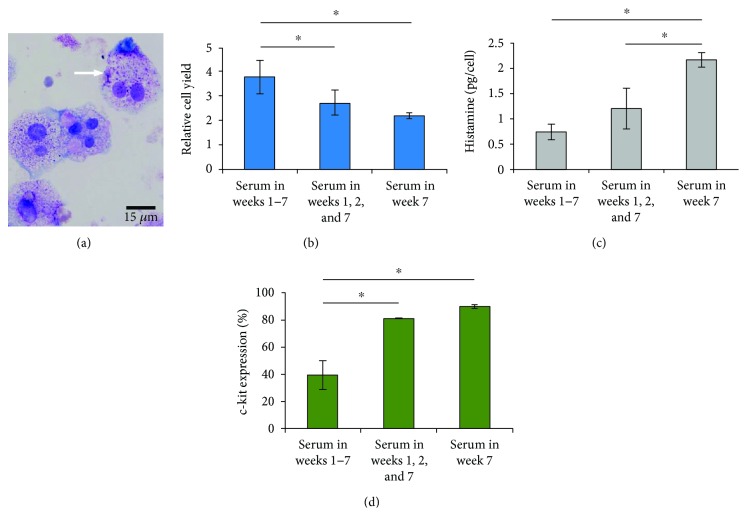
Effect of serum on cell yield, granule formation, and marker expression of the cells generated from CD133^+^ hematopoietic stem cells (HSCs) in a matrix after seven weeks in culture. (a) Metachromatic staining of cytoplasmic granules in the cells generated with serum added in the first two and last weeks of culture. Some of the generated cells were bi- or multinucleated, as highlighted by a white arrow. (b) Cell yield of generated cells with serum added at different time points. The ratio of cells collected at the end of the culture period to the cells seeded at the beginning of culture was reported as relative cell yield. (c) Histamine granule formation in the generated cells. (d) Expression of c-kit by generated cells. In all cases, StemSpan medium was used. Data are represented as mean ± SD; *n* = 3. ^∗^ indicates *p* < 0.05 between the media tested.

Our results show that the addition of serum to the culture media affected various characteristics of the HSC-derived cells, such as morphology, proliferation of HSCs, cell surface marker expression, and histamine content of the generated cells (Figures 1–3). Other studies have shown that MCs in serum-supplemented media had multilobed nuclei or macrophage-like morphology, in contrast with the cells in serum-free media [20, 56]. The results from our work also indicate that the timing of adding serum to the culture media can affect the morphology and internal structure of the generated cells, which could be related to the stage of cellular development. When serum was added in the first two and last weeks of culture, more cells had bi- (30%) or multinucleated (10%) morphology in comparison to when serum was added only in the last week of culture, with 8% and 2% bi- and multinucleated cells, respectively (Figure 3(a)). Furthermore, similar to other serum-supplemented cell cultures [57, 58], the proliferation of progenitor cells increased (by 72% for StemSpan medium) when serum was added from the beginning of culture, compared to the last week, for a seven-week culture period (*p* < 0.05, Figure 3(b)). The results indicate that although serum induces cell proliferation, the addition of serum in the beginning of culture can delay the morphologic change from promastocytes to mature MCs.

Moreover, serum appeared to have an inhibitory effect on histamine content. For MCs generated in StemSpan and StemPro, the histamine content was more than two-fold higher when serum was added in the last week of culture, compared to the beginning of culture (*p* < 0.05, Figure 1(c)). As shown in Figure 3(c), when serum was excluded from the culture media after the second week, the inhibitory effect of the serum on the formation of histamine granules was mitigated.

As shown in Figure 3(d), compared to the samples that contained serum throughout culture, excluding serum after the second week enhanced the c-kit expression, verifying that serum suppressed MC development. Although the effect of serum on Fc*ε*RI expression was not significant (*p* > 0.05), there was a significant increase in the receptor density (1.6 ± 0.2-fold, *p* < 0.05) on the surface of cells cultured in StemSpan (Ser1, 2, and 7) compared to StemSpan (Ser1–7). These results show that not only does serum affect the cellular immunophenotype, but the timing of its addition can also alter the expression and the density of the cell surface receptors. Previous studies have shown that the addition of serum to the media from the beginning of culture reduces the expression or the density of c-kit, while increasing the expression of myeloid markers (such as CD14, CD11b, and CD13) [20, 56]. These studies suggested that serum factors can induce the generation of other cell types and delay the development of MCs. However, a serum-free media could also result in lower Fc*ε*RI expression and abolish MC activation [20].

### 3.3. Effect of Culture Media on Fibroblasts

**Figure 4:**
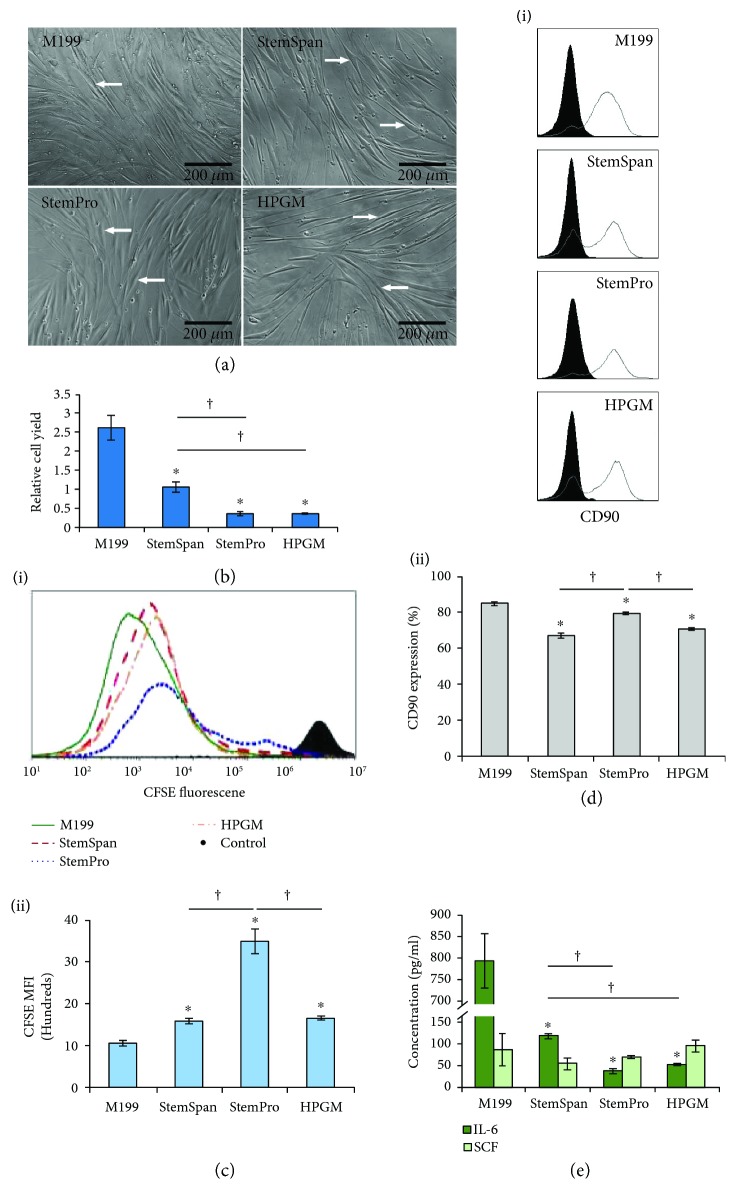
Effect of serum-free media on fibroblast growth and characteristics. (a) Morphology of fibroblasts at six days postseeding in serum-free media. Arrows show some of the typical fibroblasts. The rest of the experiments were performed ten days postseeding. (b) Fibroblast yield in the culture media. The ratio of the number of collected cells to the number of seeded cells is shown as relative cell yield. (c, (i) and (ii)) Proliferation of fibroblasts in the culture media. Solid histogram shows the cells analyzed on the day of seeding (nondividing cells). (d, (i) and (ii)) Expression of CD90 by fibroblasts in the culture media. Expression of CD90 (open histogram) was compared to an isotype control (solid histogram). (e) Release of SCF and IL-6 by fibroblasts. In all cases, serum-supplemented M199 was taken as the standard cell culture medium. Data are represented as mean ± SD; *n* = 3. ^∗^ indicates *p* < 0.05 between M199 and the test media. † indicates *p* < 0.05 between the test culture media.

As shown in Figure 4(a), fibroblasts displayed normal, elongated morphology in all the test media. The test media are typically not used to culture fibroblasts, but our results indicate that the cells show normal morphology in all the media.

The cell yields varied across the test media (Figure 4(b)). StemSpan showed a higher cell yield (more than two-fold) than StemPro and HPGM (*p* < 0.05, Figure 4(b)). As shown in Figure 4(c), (i), cells in all the media divided at least once in comparison with the control, nondividing cells. The MFI of the CellTrace CFSE-stained cells in the test media were higher than M199 (*p* < 0.05), showing lower proliferation. StemPro did not support the proliferation of fibroblasts as much as the other test media, as specified by the higher MFI of stained cells (*p* < 0.05, Figure 4(c), (ii)). Therefore, fibroblasts were more proliferative and higher in number in StemSpan than other media tested.

Fibroblasts cultured in all the test media expressed CD90 (a phenotypic marker of fibroblasts [59]), as shown in Figure 4(d), (i) and (ii). CD90 expression was higher for fibroblasts in StemPro compared to StemSpan and HPGM (*p* < 0.05). Before seeding, almost all fibroblasts were positive for CD90. The lower expression after culture shows that the culture media affected CD90 expression, which has been shown to be related to their phenotypic heterogeneity [60].

The release of the cytokines IL-6 and SCF, which are involved in the differentiation of HSCs to MCs, was measured for fibroblasts. As shown in Figure 4(e), the fibroblasts released IL-6 in a media-dependent manner, with more than two-fold higher release for the cells cultured in StemSpan, compared to the release from fibroblasts cultured in StemPro and HPGM (*p* < 0.05). The expression of SCF gene by fibroblasts has been shown in previous studies [61] and in this work, SCF was released similarly by fibroblasts in all the test media (Figure 4(e)). These data show that fibroblasts can release growth factors, which are necessary for the development and survival of MCs. The concentrations of IL-6 and SCF in the fibroblast culture media were not as high as the concentrations in the HSC media used for MC generation. However, the coculture of fibroblasts with MCs has been shown to upregulate the IL-6 release [62] and may have the same effect on SCF secretion. Besides the effect of soluble factors in the medium, the direct interaction of fibroblasts with MCs was reported to be necessary for MC maturity, inhibition of apoptosis, and generation of MC subtypes specifically found in the connective tissue [63, 64]. Overall, considering the results from testing all of the media, StemSpan was superior for fibroblast culture in a stem cell media.

### 3.4. Effect of Culture Media on Endothelial Cells (ECs)

**Figure 5:**
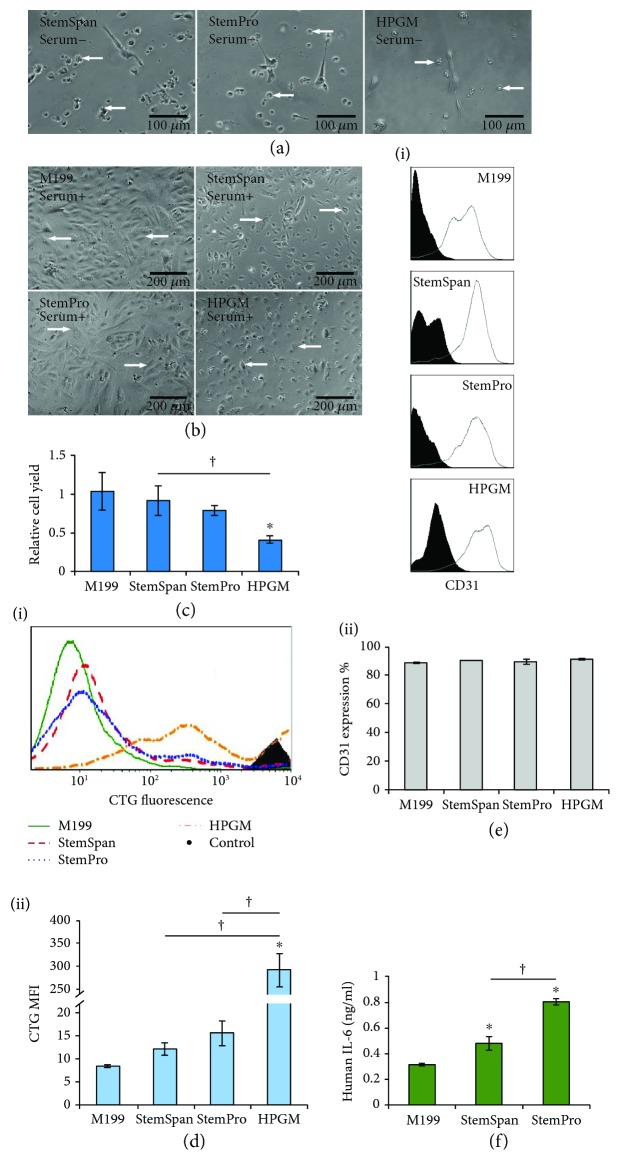
Effect of culture media on endothelial cell (EC) growth and characteristics. (a) Micrographs depicting poor survival of ECs at three days postseeding in serum-free media. Typical detached or dead ECs are highlighted by arrows. (b) Micrographs depicting survival of ECs at six days postseeding in serum-supplemented media. Typical cells are highlighted by arrows. The rest of the experiments were performed 12 days postseeding. (c) EC yields in the serum-supplemented media. Ratio of the number of collected cells to the number of seeded cells is shown as relative cell yield. (d, (i) and (ii)) Proliferation of ECs in the serum-supplemented media. Solid histogram shows the nondividing cells. (e, (i) and (ii)) Expression of CD31 by ECs in the serum-supplemented media. The expression of CD31 (open histogram) was compared to an isotype control (solid histogram). (f) Release of IL-6 by ECs. In all cases, serum-supplemented M199 was taken as the standard cell culture medium. Data are represented as mean ± SD; *n* = 3. ^∗^ indicates *p* < 0.05 between M199 and test media. † indicates *p* < 0.05 between test culture media.

In contrast with fibroblasts, ECs failed to survive beyond three days in any of the serum-free media (Figure 5(a)). As shown in Figure 5(b), for the serum-supplemented media, ECs showed characteristic, cobblestone-like morphology. The cell yield of ECs was the lowest in HPGM among all the media tested, shown in Figure 5(c). When measuring cell proliferation, the ECs divided at least once in all the culture media in comparison with the control, nondividing cells (solid histogram, Figure 5(d), (i)). ECs cultured in HPGM showed the highest MFI among the other test media, indicating the lowest cell proliferation (*p* < 0.05, Figure 5(d), (ii)). Therefore, HPGM did not support the survival and proliferation of ECs to the extent seen with the other culture media.

The EC phenotypic marker, CD31 [65], was highly expressed for all cells in all the media tested (Figure 5(e), (i) and (ii)), indicating that culture media did not affect the typical EC phenotype.

The release of IL-6 and SCF was also measured for ECs. The release of IL-6 from ECs cultured in StemPro was higher than ECs cultured in StemSpan (*p* < 0.05, Figure 5(f)) and it was not detected from ECs cultured in HPGM (with the ELISA detection limit of 24 pg/ml). Higher levels of IL-6 in StemSpan and StemPro compared to the control media (M199) might be the effect of the media contents or supplements. IL-6 is a cytokine that facilitates the survival and maturation of MCs [66, 67]. In addition to the effect of soluble factors, the direct interaction of adhesion molecules on ECs and c-kit receptors on MCs have been shown to regulate MC survival and the development of connective tissue-type MCs [27]. Although, previous studies have reported the expression of the SCF gene by ECs [68], SCF was not detected in any of the media tested (with the ELISA detection limit of 16 pg/ml). Other studies have shown that SCF was either not released from ECs, or was released at low levels (e.g., 24.5 ± 1.5 pg/ml) [27, 69]. Taken together, our data suggests that both StemSpan and StemPro supplemented with serum supported EC proliferation and characteristic phenotype.

### 3.5. Mast Cell (MC) Morphological Phenotype and Function

**Figure 6:**
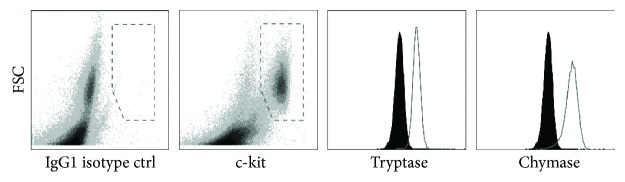
Expression of tryptase and chymase by generated cells from CD133^+^ hematopoietic stem cells (HSCs) in a collagen matrix after seven weeks in culture. StemSpan with serum in the seventh week of culture was used as culture media. Representative density plots and histograms of the marker expression are shown. Expression of tryptase and chymase by c-kit-positive cells (gray histogram) is compared with the isotype control (black histogram).

Considering all the media tested for this study, and specifically focusing on histamine content and immunophenotype of the generated cells, we believe that StemSpan with serum added in the last week of culture is the most suitable media for MC development from HSCs. Also, StemSpan supported the ancillary cells. Therefore, the morphological phenotype and function of the cells generated within the matrix in this media after seven weeks in culture were examined. Human MCs are heterogeneous and on the basis of the expression of serine proteases have been classified to tryptase-positive (MC_T_), chymase-positive (MC_C_), and tryptase- and chymase-positive (MC_TC_) MCs [1]. As shown in Figure 6, almost all the generated c-kit-positive cells were expressing tryptase (99.5 ± 0.2%) and chymase (97.1 ± 0.9%) granules, exhibiting the MC_TC_ phenotype predominant in the skin and small intestinal submucosa [23]. However, when StemSpan was used to generate MCs from CD133^+^ HSCs in a 2D culture system, the MC_T_ subtype was observed [16]. This highlights the influence of the microenvironments or culture conditions on the MC phenotype.

**Figure 7:**
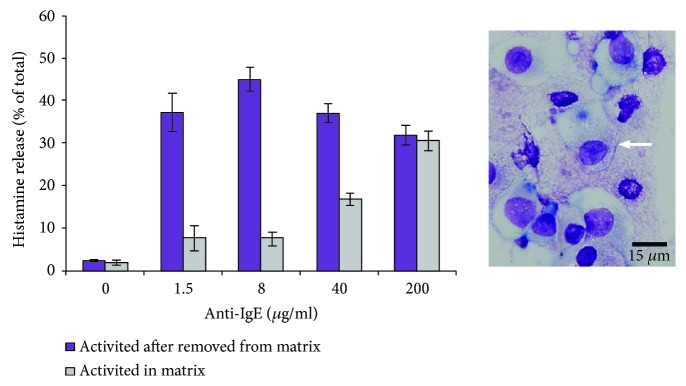
Histamine release by the generated mast cells (MCs) after seven weeks in culture. The cells were activated either within the matrix or after being removed from the matrix. The micrograph shows a few of the degranulated cells activated within the matrix. The white arrow highlights a degranulated MC. In all cases, StemSpan medium with serum in the seventh week of culture was used. Data are represented as mean ± SD; *n* = 3. All the data are significantly higher than the nonactivated samples (*p* < 0.05).

Histamine is one of the vasoactive amines in MC granules released upon activation. Sensitization with IgE and activation of the cells with various concentrations of anti-IgE induced substantial degranulation of the cells upon Fc*ε*RI cross-linking (Figure 7). The activation of cells removed from the matrix and cells in the matrix resulted in the release of up to 45 ± 3% and 35 ± 3% of the histamine content, respectively, which was about 19-fold higher than the spontaneous histamine release (*p* < 0.05). Also, Wright-Giemsa staining showed that cells partially or completely lost the metachromatic granules after activation (Figure 7).

*In vivo* MCs can release less than 10% to more than 40% of the total histamine content in response to the anti-IgE [70–72]. In this work, the generated cells released up to 48% of the histamine content in an anti-IgE concentration-dependent manner, which is comparable to the histamine release from generated MCs in a 2D culture system [15].

The response from the generated cells that were activated either after the removal from the matrix or within the matrix was different with respect to anti-IgE concentration. For the cells that were activated after removal from the matrix, the maximum response was measured for 8 *μ*g/ml anti-IgE, and any further increase of the anti-IgE concentration resulted in a decreased response. For the cells that were activated within the matrix, increasing the anti-IgE concentration up to 200 *μ*g/ml resulted in increased responses, but never reached a maximum response as seen for the cells removed from the matrix. The lower response from the cells within the matrix may be due to the matrix interfering with the binding of IgE and/or anti-IgE with the cells, either due to transport limitations and/or nonspecific binding. In fact, previous studies have shown that other proteins, like monocyte chemoattractant protein-1 (MCP-1), can bind with the matrix and establish a concentration gradient [73, 74]. Therefore, a higher concentration of the IgE antibody was needed to overcome any loss due to the matrix. Nevertheless, the results show that MCs generated within the matrix are functional and release histamine in an IgE-mediated reaction.

## 4. Conclusion

In this work, we have established that MCs can be generated from HSCs isolated from peripheral blood within a 3D collagen matrix, based on the morphology of the CD133^+^-derived cells, the formation of cytoplasmic granules (histamine), and the expression of MC phenotypic markers (especially c-kit). In addition, according to the same criteria mentioned above, StemSpan with serum added in the last week of culture was the best media to generate functional MCs. StemSpan was also suitable for fibroblast and EC culture. Therefore, we established StemSpan as the ideal media, since it supports the differentiation of HSCs to MCs and phenotypic characteristics of ancillary cells. Furthermore, we determined that serum was not required for fibroblasts, but was required for EC survival and MC maturation during the last week of culture. As a result, to develop the 3D tissue model with all three cell types, fibroblasts and CD133^+^ cells would be seeded in the collagen matrix for six weeks using the serum-free media, then ECs would be added to the apical surface of the matrix in a serum-supplemented media during the seventh week of culture. This work demonstrates the possibility of creating the tissue model that could be used to study the effect of the microenvironmental factors with ancillary cells on MC development and function. However, this study shows that under the influence of microenvironmental factors the morphological and functional characteristics of the cells generated in 3D culture conditions can be altered, as evidenced by differences in their subtype and response to an activating agent when compared with a 2D culture system. The possibility of studying the effect of microenvironmental factors can be considered as the main advantage of utilizing the 3D matrix-embedded cells in elucidating MC ontogeny, biological profile, and immunoregulatory roles.

## Data Availability

Data underlying the findings of the study is available upon request to the corresponding author.
